# Pyruvate alleviates high glucose‐induced endoplasmic reticulum stress and apoptosis in HK‐2 cells

**DOI:** 10.1002/2211-5463.12834

**Published:** 2020-04-10

**Authors:** Xiao Meng Zhang, Yi Zhen Wang, Jin Dong Tong, Xu Chao Ning, Fang Qiang Zhou, Xiu Hong Yang, Hui Min Jin

**Affiliations:** ^1^ Department of Nephrology Pudong Medical Center Shanghai Pudong Hospital Fudan University Shanghai China; ^2^ Department of Clinical Medicine Affiliated Hospital of Qingdao University Shandong China; ^3^ Division of Vascular Surgery Pudong Medical Center Shanghai Pudong Hospital Fudan University Shanghai China; ^4^ Shanghai Sandai Pharmaceutical R&D Co., Ltd. Pudong China; ^5^ Present address: US office Las Vegas NV USA

**Keywords:** apoptosis, diabetes, diabetic nephropathy, endoplasmic reticulum stress, HK‐2 cells, pyruvate

## Abstract

Endoplasmic reticulum (ER) stress plays a critical role in the development of diabetic nephropathy (DN). We previously demonstrated that pyruvate (Pyr)‐enriched oral rehydration solution improved glucometabolic disorders and ameliorated DN outcome in db/db mice. Here, we investigated the effects of Pyr on high glucose‐induced ER stress and apoptosis in HK‐2 cells. Our results suggest that high glucose can induce reactive oxygen species production, apoptosis and ER stress in HK‐2 cells, and that Pyr treatment can ameliorate these effects and restore the expression of key proteins involved in ER stress. Thus, Pyr may have potential for the development of novel strategies for the prevention and treatment of clinical DN.

## Graphical abstract

Pyruvate is a scavenger of reactive oxygen species in the presence of oxygen and during hyperglycemia, and it protects against tissue injuries of various insults. Here, we investigated the effects of pyruvate on the HK‐2 cell line under high glucose and concluded that pyruvate ameliorated endoplasmic reticulum stress and inhibited apoptosis under high glucose. Pyruvate may have potential for using in novel strategies for clinical intervention of diabetic nephropathy.

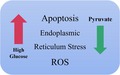

**Abbreviations**
ATF4: activating transcription factor 4
CCK‐8: Cell Counting Kit‐8
CHOP: C/EBP homologous protein
CON: control (group)
DMEM: Dulbecco’s modified Eagle’s medium
DN: diabetic nephropathy
ER: endoplasmic reticulum
GRP78: glucose‐regulated protein 78
HG: high glucose
p‐EIF2α: phosphorylate α‐subunit of eukaryotic initiation factor 2
ROS: reactive oxygen species

According to the 2019 Global Kidney Health Atlas (GKHA editions), nephritic disease is a serious clinical and public health problem, and has become an increasingly tremendous burden for individuals, society and medical care [1]. Diabetic nephropathy (DN) is the leading cause of chronic kidney disease in western countries. Lack of effective treatments has increased the challenge for the diabetic community worldwide [2].

Extensive research has shown that endoplasmic reticulum (ER) stress‐induced apoptotic pathways are frequently described in many diseases. ER stress plays a critical role in the progress of diabetes and DN [3, 4]. It is a series of pipeline systems composed of membranes in the cytosol and is a critical cell organelle with multiple functions. ER has the function of folding, modifying and degrading secretary membrane‐bound proteins. In the pathological state, the ER stress is activated and leads to unfolded protein response. Mild ER stress does not damage cells, but long‐term ER stress causes apoptosis [5]. Activation of the ER stress has been repeatedly demonstrated to damage cells, such as islets, nerves and renal tubules, in the presence of high glucose (HG), which is one of the important pathological processes of the diseases [6, 7, 8].

Pyruvate (Pyr) is an important product of glycolysis, which can scavenge reactive oxygen species (ROS) in the presence of oxygen and hyperglycemia, and protect against tissue injury. Accumulating animal experiments and some clinical studies have convincingly substantiated that exogenous Pyr is protective from glucose metabolic defects in various pathogenic insults (e.g., hypoxia/ischemia, hemorrhage, trauma/burn and even sepsis) in the past several decades [9, 10, 11]. It has been ascertained that Pyr is of unique pleiotropic pharmacological characteristics: enhancement of hypoxia tolerance, correction of disturbances of glucose metabolism and acid–base balance, exertion of antioxidative stress and inflammation, preservation of mitochondrial structure and function, and prevention from cellular apoptosis [12, 13, 14]. Further, case reports showed that oral Pyr markedly attenuated diabetic status in patients with type 1 diabetes, and Pyr stimulated insulin secretion in a patient with diabetes [15, 16]. However, Pyr effects on HG‐induced ER stress and apoptosis in HK‐2 cells remain unexplored.

This research was undertaken to focus on Pyr effects on ER stress and cellular apoptosis in HK‐2 cells, as one of the molecular mechanisms of DN onset and progression. Our results showed that Pyr ameliorated the ER stress and inhibited apoptosis in HK‐2 cells under HG. Pyr may provide a possibility in the treatment of clinical diabetes and DN as a novel strategy.

## Materials and methods

### Cell culture

HK‐2 cells, a line of human renal proximal tubular epithelial cells, were purchased from Fuheng Cell Center (Shanghai, China). HK‐2 cells were maintained in Dulbecco’s modified Eagle’s medium (DMEM) without Pyr (Gibco, Carlsbad, CA, USA) supplemented with 10% FBS (Hyclone, Logan, UT, USA) and 2% penicillin–streptomycin solution (Gibco) at 37 °C, 5% CO_2_.

### Cell viability assay by Cell Counting Kit‐8

HK‐2 cell viability was detected by Cell Counting Kit‐8 (CCK‐8) assay (Dojindo, Tokyo, Japan). In brief, HK‐2 cells were seeded into 96‐well plates and exposed to 30 mm glucose and various doses of Pyr from 0.01 to 10 mm (final concentrations) for 3 or 4 days. After the treatments, 10 μL CCK‐8 solution was added into each well, and the cells were additionally cultured at 37 °C for 3 h to check cell viability. Data were obtained by microplate reader (Infinite M200 pro; Tecan Inc., Männedorf, Switzerland) at 450 nm absorbance (*A*_450 nm_) [17].

### Flow cytometry analysis

Annexin V–FITC/propidium iodide (PI) apoptosis assay kit (BD, Franklin Lake, NJ, USA) was used to analyze the apoptosis rate by flow cytometry. In brief, cells were grown to about 70% confluence in six‐well plates and treated with 30 mm HG and/or 0.5 mm Pyr for 72 h. Cells in each sample were washed twice with PBS and then resuspended in 1× Binding Buffer. Thereafter, the suspension (1 × 10^5^ cells) was transferred to a 5‐mL culture tube, and Annexin V–FITC and PI were added to each culture tube. Finally, cells were measured by a flow cytometer (BD). Besides, ROS levels were detected by a fluorometric assay, using the 2′,7′‐dichlorodihydrofluorescein diacetate (DCFH‐DA; Sigma Aldrich, St. Louis, MO, USA) as a fluorescence probe [18]. Sodium pyruvate (Pyr) was purchased from Sigma Aldrich.

### Western blotting

The lysis buffer (Invent, Eden Prairie, MN, USA) was used to extract the proteins. Proteins were separated and transferred onto poly(vinylidene difluoride) membrane. In brief, the protein was detected as described previously [19]. The primary antibodies against Bcl‐2, BAX, Caspase‐3, glucose‐regulated protein 78 (GRP78), C/EBP homologous protein (CHOP), activating transcription factor 4 (ATF4) and phosphorylate α‐subunit of eukaryotic initiation factor 2 (p‐EIF2α) and the secondary antibodies (anti‐mouse IgG, anti‐rabbit IgG) were obtained from Proteintech Group, Inc (Chicago, IL, USA), and the antibody against p‐EIF2α was purchased from CST (Danvers, MA, USA). Proteins brands were quantified and analyzed with imagej software (University of Wisconsin, Madison, WI, USA).

### Statistical analysis

Results were presented as the mean ± standard error of the mean (SEM). Statistical analyses were performed using spss 22.0 statistical software (SPSS, Chicago, IL, USA). The group differences were calculated by one‐way ANOVA; *P* < 0.05 was considered statistically significant.

## Results

### Pyr increased cell viability under HG

**Fig. 1:**
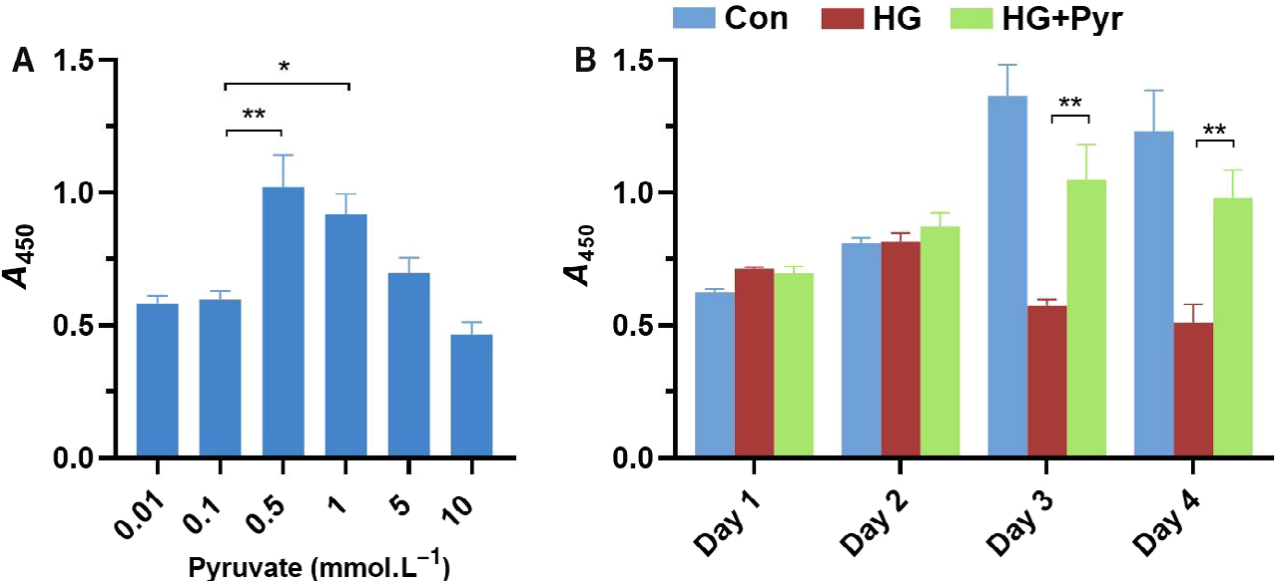
Pyr effects on cell proliferation as evaluated by CCK‐8. (A) HK‐2 cells were exposed to HG (30 mm) and treated with 0.01–10 mm Pyr for 72 h. (B) HK‐2 cells were exposed to HG (30 mm glucose) and treated with Pyr (0.5 mm) for 4 days. Con: cells were treated in 5 mm glucose in the DMEM. Values were means ± SEM (*n* = 5). Data were analyzed by one‐way ANOVA. *P < 0.05; **P < 0.01.

CCK‐8 assay showed the cell viability. As shown in Fig 1A, other than at 0.5 and 1.0 mm, pyruvate at 0.01, 0.1, 5 and 10 mm, had no notable protective effects on the cell viability under 30 mm glucose in HK‐2 cells. Cell viability was the highest on the days 3–4 in cell cultures (Fig. 1B). Pyr might be not cytoprotective at or greater than 5 mm in cell proliferation. HG decreased cell viability; however, Pyr pretreatments significantly attenuated the detrimental effect of HG on cell viability in human HK‐2 cells. According to the data in Fig. 1B, HK‐2 cells had the highest cell viability when the Pyr concentration was 0.5 mm on the third day; thus, it was revealed that exogenous Pyr at 0.5 mm had significantly cytoprotective function.

### Pyr inhibited HG‐induced apoptosis in HK‐2 cells

**Fig. 2:**
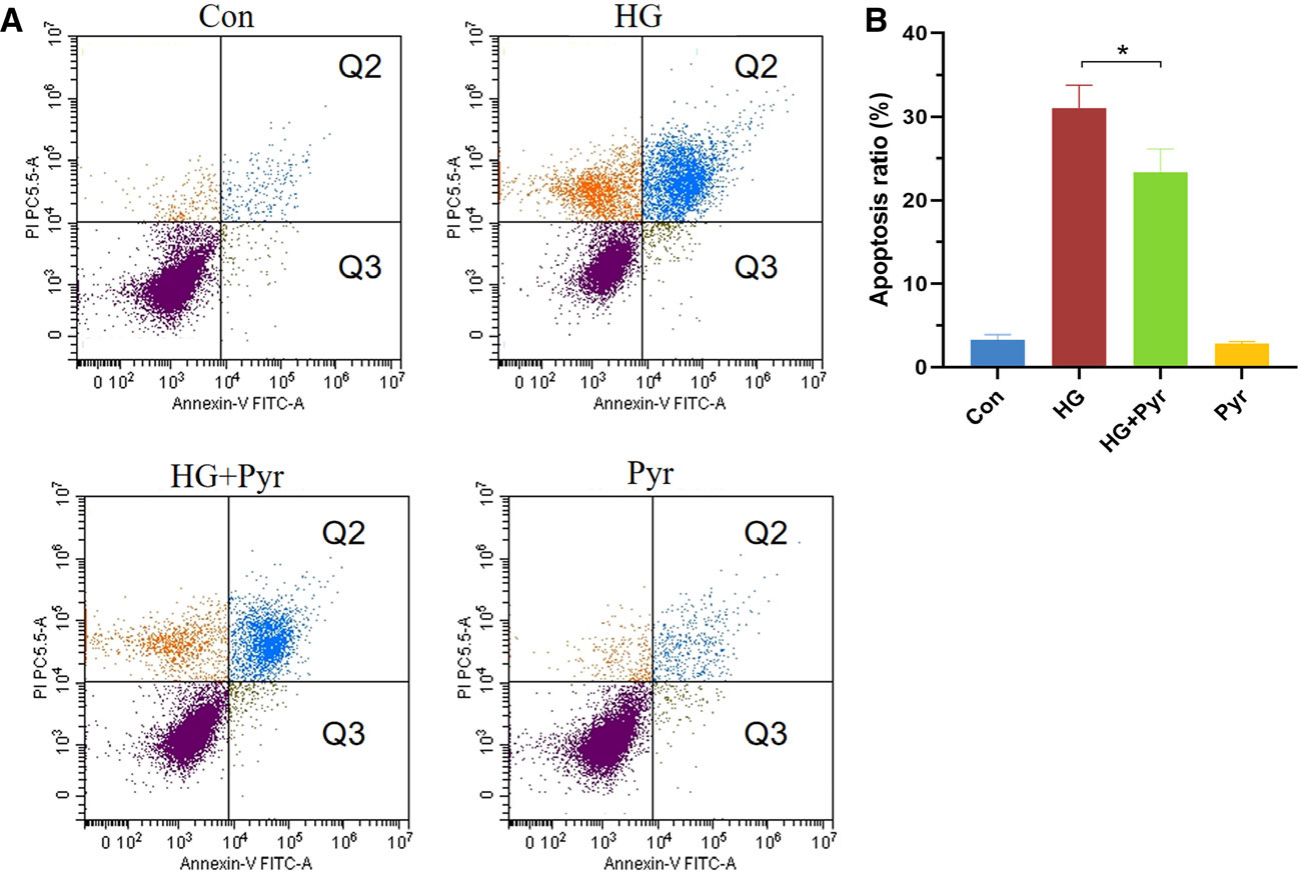
Pyr effects on cell apoptosis by flow cytometry analysis. HK‐2 cells were exposed to HG and treated with Pyr (0.5 mm) for 72 h. Cells were stained with Annexin V/PI for flow cytometry analysis (A). The Q2 (Annexin V–FITC^+^/PI^+^) and Q3 (Annexin V–FITC^+^/PI^−^) were considered as early stage and late stage of apoptotic cells, respectively. Thus, the apoptosis ratio (B) was quantified by Q2 + Q3. The apoptosis ratio was expressed in a histogram. Con: cells were treated with 5 mm glucose in the DMEM; HG: cells were treated with 30 mm glucose in the DMEM; HG + Pyr: cells were treated with 30 mm glucose and 0.5 mm Pyr in the DMEM; Pyr: cells were treated with 5 mm glucose and 0.5 mm Pyr in the DMEM. Values are represented as mean ± SEM (*n* = 5). Data were analyzed by one‐way ANOVA. *P < 0.05.

As shown in Fig. 2A, HK‐2 cells were treated with or without HG (30 mm) and Pyr (0.5 mm) for 3 days. Compared with the HG group, apoptosis ratios of the HG + Pyr group (Fig. 2B) were significantly decreased by flow cytometry analysis after Pyr treatments. Group Pyr had no difference in the apoptosis ratio from the control group (group Con). These results demonstrated that exogenous Pyr at 0.5 mm inhibited HK‐2 cells apoptosis under HG.

### Pyr restored apoptosis‐related proteins

**Fig. 3:**
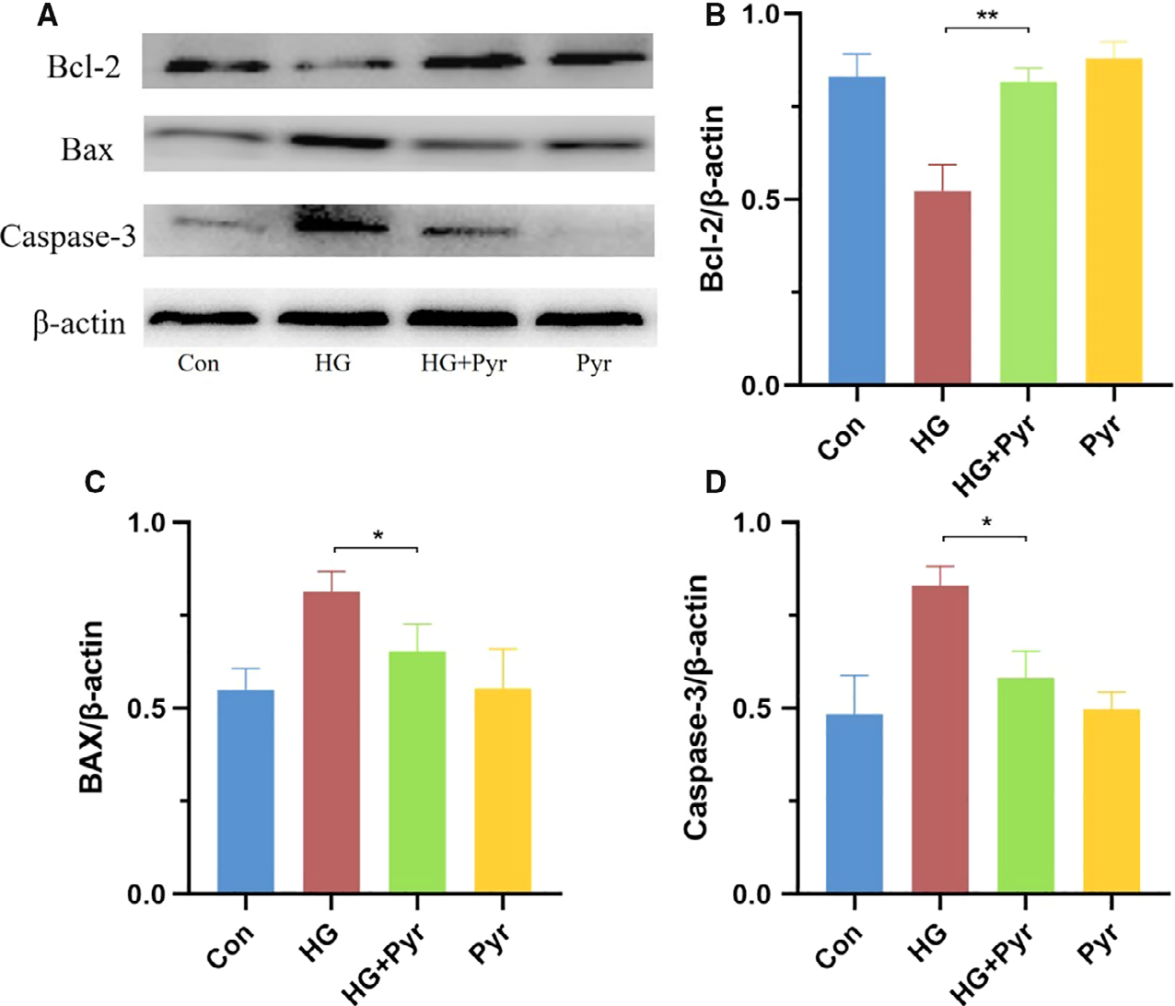
Pyr effects on apoptosis‐related protein expressions. HK‐2 cells were exposed to HG (30 mm glucose) and treated with Pyr (0.5 mm) for 72 h. (A) The protein expressions of Bcl‐2, BAX and Caspase‐3 of HK‐2 cells after exposure to HG in the presence or absence of Pyr were detected by western blot analyses. Con: cells were treated with 5 mm glucose in the DMEM; HG: cells were treated with 30 mm glucose in the DMEM; HG + Pyr: cells were treated with 30 mm glucose and 0.5 mm Pyr in the DMEM; Pyr: cells were treated with 5 mm glucose and 0.5 mm Pyr in the DMEM. The percentages of Bcl‐2 (B), BAX (C) and caspase‐3 (D)/β‐actin in the bar graphs were quantified by imagej software. Values were means ± SEM (*n* = 5). Data were analyzed by one‐way ANOVA. *P < 0.05; **P < 0.01.

Data regarding apoptosis‐related proteins (Bcl‐2, BAX and Caspase‐3) were shown in Fig. 3A. The expressions of BAX (Fig. 3C) and Caspase‐3 (Fig. 3D) were upregulated, but the level of Bcl‐2 (Fig. 3B) was downregulated in HK‐2 cells under HG. With appropriate Pyr in HK‐2 cells, as illustrated in Fig. 3B–D, exogenous Pyr fully restored the Bcl‐2 and inhibited BAX and Caspase‐3 expressions.

### Pyr restored expressions of ER stress‐related proteins

**Fig. 4:**
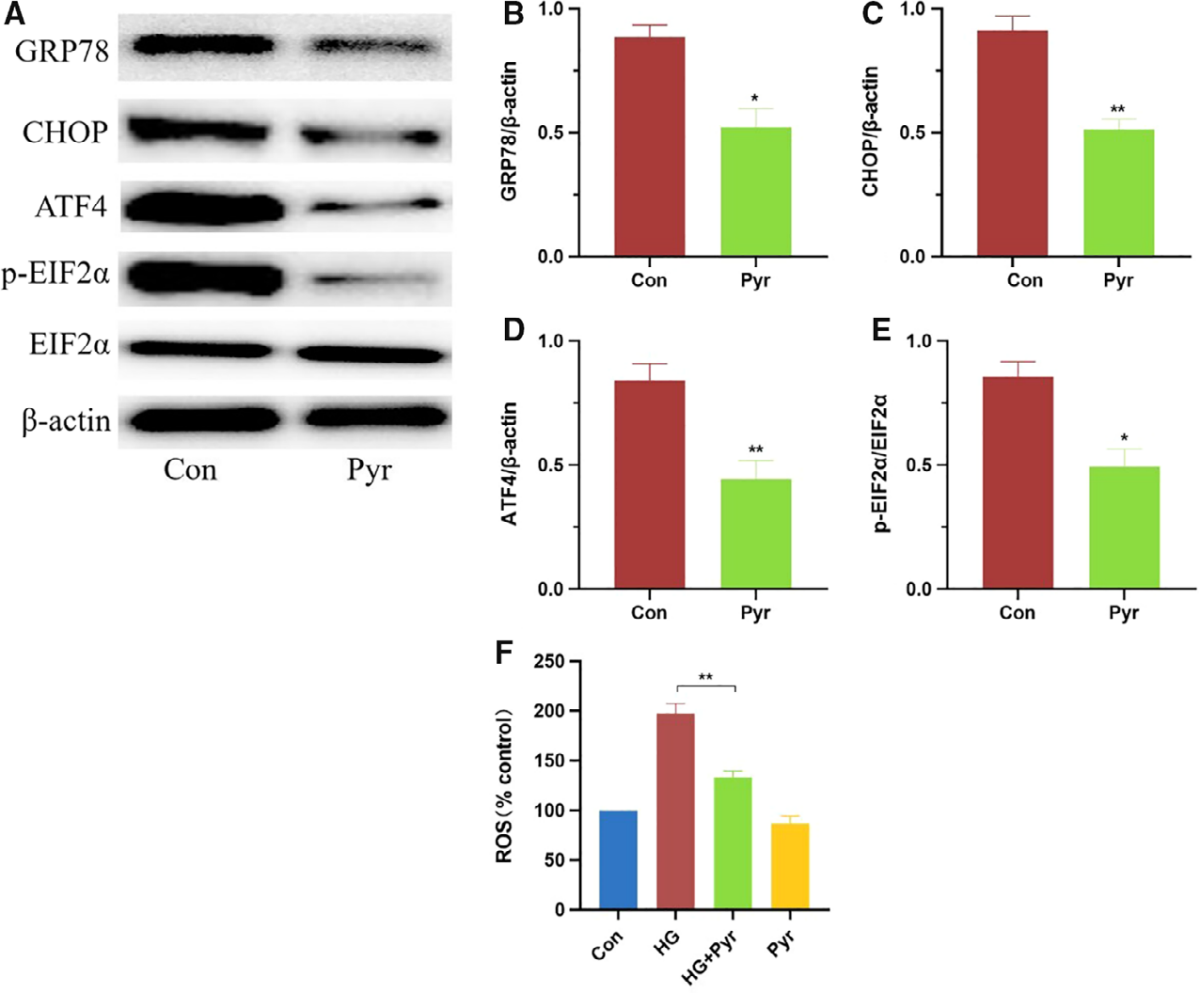
Pyr effects on ER stress‐related protein and ROS level. HK‐2 cells were treated with Pyr (0.5 mm) and/or HG (30 mm glucose) for 3 days. The protein expressions (A) of GRP78, CHOP, ATF4 and p‐EIF2α of HK‐2 cell were detected by western blot analyses. The percentages of GRP78 (B), CHOP (C), ATF4 (D) and p‐EIF2α (E)/β‐actin in the bar graphs were quantified by imagej software. Expressions of GRP78 (B), CHOP (C), ATF4 (D) and p‐EIF2α (E) were decreased after Pyr treatments, which ascertained that exogenous Pyr ameliorated the ER stress in HK‐2 cells. (F) HK‐2 cells were treated with Pyr (0.5 mm) and/or HG (30 mm glucose) for 3 days. Further, HG‐induced ROS increases were inhibited by Pyr treatments in HK‐2 cells. Con: cells were treated with 5 mm glucose in the DMEM; HG: cells were treated with 30 mm glucose in the DMEM; HG + Pyr: cells were treated with 30 mm glucose and 0.5 mm Pyr in the DMEM; Pyr: cells were treated with 5 mm glucose and 0.5 mm Pyr in the DMEM. Values were means ± SEM (*n* = 5). Data were analyzed by one‐way ANOVA. *P < 0.05; **P < 0.01.

In Fig. 4A, the protein expressions of GRP78, CHOP, ATF4 and p‐EIF2α in group Pyr were significantly decreased compared with group Con in HK‐2 cell. GRP78 (Fig. 4B), CHOP (Fig. 4C), ATF4 (Fig. 4D) and p‐EIF2α (Fig. 4E) were classic marker proteins of ER stress. After quantification by imagej software, expressions of GRP78 (Fig. 4B), CHOP (Fig. 4C), ATF4 (Fig. 4D) and p‐EIF2α (Fig. 4E) were significantly decreased after Pyr treatments, which ascertained that exogenous Pyr ameliorated the ER stress in HK‐2 cells.

### Pyr ameliorated ROS production

In Fig. 4F, considering a relationship between ER stress and ROS [20], the ROS level was further examined. It was found that the ROS level in the HG group was significantly increased, as compared with the normal glucose group. However, with suitable Pyr, the ROS level was significantly decreased in the group HG + Pyr with no difference between group Con and group Pyr. The results evidenced that exogenous Pyr treatment ameliorated ROS formation under HG.

## Discussion

Sodium Pyr has been experimentally proved to improve glucose metabolism and renoprotection [21, 22]. Present experiments further explored the protective effect of Pyr on HK‐2 cells under the HG condition. There are some aberrant factors causing renal injury in diabetes, such as abnormal glucose metabolism with Warburg phenomenon and dysfunction of mitochondria in glomerular endothelial and tubular epithelial cells, as well as advanced glycation end products [2, 23]. In recent studies in diabetes, ER stress has been a major area of interest [24]. In addition, ER stress is associated with a variety of diseases, such as vascular diseases, neurodegenerative diseases and cancer [6, 25, 26]. Numerous studies established that GRP78 is a marker for ER stress. GRP78 is a central regulator for ER stress [27]. Within the normal range of glucose, GRP78 can combine with other ER stress factors and keep in an inactive state. On the contrary, overexpression of GRP78 in HG leads to cell death [20]. CHOP is downstream of ATF4, a key protein in the ER stress pathway. Both CHOP and ATF4 are transcription factors that regulate unfolded protein response target genes. Under pathological conditions, ATF4 is a key proapoptotic factor that dephosphorylates EIF2α [28, 29].

The ER pathway activation has been convincingly substantiated to injure various cells, including renal tubules epithelium in the presence of HG [6, 7, 8]. The present research first explored whether the effects of proper Pyr might robustly decrease expressions of these ER proteins in HG, which would be otherwise activated in the ER stress pathway and cause cell death (Fig. 4A–E).

Chronic ER stress caused by unrelenting internal or external insults, including ROS, produces a secondary increase in ROS, generally resulting in cell death [30]. Long‐term HG leads to the dysfunction of cells, which may directly induce kidney injury and excessive production of ROS. With the increased ROS levels, cells lead to disruption of normal cell physiology, such as lipid peroxidation, DNA modification, protein misfolding, impairment of antioxidant system and mitochondrial damage, leading to cellular apoptosis and ferroptosis [31, 32]. In other studies, it was also mentioned that Pyr reduced the oxidative stress of diabetic eye diseases in rodents [33]. The results in this experiment, *in vitro*, demonstrated that ROS production was decreased after the addition of proper Pyr (Fig. 4F). Our data also suggested a possibility that Pyr suppressed ER stress probably via additional inhibition of ROS generation.

HG, HG‐induced ROS and resultant ER stress are closely related to apoptosis [34]. Apoptosis‐related proteins, BAX/Bcl‐2 family and Caspase‐3, play an important role in regulating the process of apoptosis. Previous studies have found that Pyr can inhibit cellular apoptosis in ischemia‐reperfusion injury and diabetic eye diseases [12, 21, 35]. The present data confirmed that Pyr inhibited HK‐2 cell apoptosis under HG (Figs 2 and 3). These results, for the first time, demonstrated that sodium Pyr in suitable levels had the alleviative effect of ER stress, ameliorating ROS production, inhibiting apoptosis and increasing cell viability in HK‐2 cells with HG. The earlier favorable results were further supported with promising findings that Pyr reactivated the Pyr dehydrogenase activity inhibited by HG with HK‐2 cells in the identical experimental conditions (data not shown). However, the underlying molecular mechanisms of Pyr inhibition of ER stress in HG required further studies.

It was indicated in Fig. 1B that the maximal Pyr protection from cell damage in HG appeared on the third day, and the protective effect might persist in the following few days *in vitro*. Therefore, continuous use of Pyr daily may sustain the beneficial effect for a long period. Notably, investigations here showed that 0.5 mm Pyr provided the best protection from HG‐induced ER stress in HK‐2 cells (Figs 1A and 4). Comparably, the blood Pyr level was increased to more than five times the normal Pyr level (0.1 mm) after enteral Pyr in Pyr‐enriched oral rehydration solution in rats subjected to severe burn injury [35], which was consistent with our findings with oral Pyr renoprotection, including Pyr dehydrogenase reactivation in diabetic db/db mice (XM Zhang, H Deng, JD Tong, YZ Wang, XC Ning, XH Yang, F‐Q Zhou, HM Jin, data submitted for publication). Accordingly, our results, *in vitro*, support that oral Pyr in Pyr‐enriched oral rehydration solution may benefit in diabetes treatment *in vivo*. In addition, excessive production of ROS and ER stress is one of the processes of various diseases. Thus, the present studies indicate that Pyr may be also applicable to other diseases besides diabetes.

Finally, ethyl Pyr (a derivative of sodium salt of Pyr) was recently shown to have an inhibitive effect on ER stress, *in vivo*, in a rat model [36], which strongly supported the present findings, *in vitro*, but ethyl Pyr does not work in humans [37].

In conclusion, Pyr alleviates HG‐induced ER stress and cellular apoptosis in HK‐2 cells, which may highlight one of molecular mechanisms in Pyr treatment of diabetes and DN progression. Our data provide an additional possibility that Pyr may be a new strategy for the clinical DN treatment in the future.

## Conflict of interest

The authors declare no conflict of interest.

## Author contributions

FQZ, XHY and HMJ designed experiments. XMZ, JDT, XCN and YZW performed experiments, corrected data and conducted statistical analysis. FQZ, XHY, XMZ and HMJ wrote the manuscript drafts. HMJ and FQZ critically revised the manuscript, and all authors have approved the final version of the manuscript and have agreed to submit it to this journal. XMZ, YZW and JDT contributed equally to this paper.
